# Vacancy‐Induced Z‐Contrast Anomaly in Self‐Assembled (Ti,V)O_2_ Heterostructure

**DOI:** 10.1002/smll.202513789

**Published:** 2026-03-30

**Authors:** Hyeji Sim, Seung‐Hyun Heo, Gyung‐Min Park, Gi‐Yeop Kim, Jaeseoung Park, Junwoo Son, Si‐Young Choi

**Affiliations:** ^1^ Department of Materials Science and Engineering Pohang University of Science and Technology (POSTECH) Pohang Republic of Korea; ^2^ Next‐Generation Semiconductor Device Research Section Electronics and Telecommunications Research Institute (ETRI) Daejeon Republic of Korea; ^3^ Department of Materials Science and Engineering Seoul National University Seoul Republic of Korea; ^4^ Research Institute of Advanced Materials Seoul National University Seoul Republic of Korea; ^5^ Center of Van der Waals Quantum Solids Institute For Basic Science (IBS) Pohang Republic of Korea; ^6^ Department of Semiconductor Engineering Pohang University of Science and Technology (POSTECH) Pohang Republic of Korea

**Keywords:** de‐channeling, oxygen vacancies, scanning transmission electron microscopy, self‐assembled heterostructure, vanadium dioxide

## Abstract

Annular dark‐field scanning transmission electron microscopy (ADF‐STEM) imaging is often called as the Z‐contrast, where the element with a higher atomic number gives rise to the brighter contrast. ADF‐STEM imaging on the self‐assembled (Ti,V)O_2_ heterostructure, exhibiting the alternating V‐rich and Ti‐rich layers, unexpectedly reveals the higher contrast in V‐rich layers in spite of the nearly identical atomic numbers of Ti (*Z* = 22) and V (*Z* = 23). Our analyses of local strain mapping and electron energy loss spectroscopy (EELS) confirm that oxygen vacancies are formed dominantly in the V‐rich and thus the higher contrast in the V‐rich layers is attributable to the accumulation of oxygen vacancies facilitated by the inherent [001] channel pathways of the rutile structure. Our results highlight the critical role of crystallographic pathways in guiding oxygen vacancy diffusion within rutile‐based heterostructures.

## Graphical abstract

This study investigates the unexpectedly high contrast observed in ADF‐STEM imaging of spinodal decomposed (Ti,V)O_2_ heterostructure. In situ heating, GPA, and EELS analyses reveal that oxygen vacancies accumulate in V‐rich layers due to facilitated vacancy migration along [001]_R_ pathways during phase separation by spontaneous ionic diffusion. These findings provide insights into defect engineering in correlated oxide heterostructure systems.

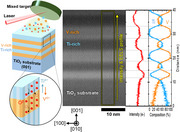

## 1 Introduction

Annular dark‐field scanning transmission electron microscopy (ADF‐STEM) is a powerful technique for probing atomic and chemical structures of crystalline materials at atomic resolution [1, 43, 46]. ADF detectors collect incoherently scattered electrons resulting from interactions with atomic nuclei, enabling intuitive visualization of atomic arrangements: empty spaces appear as dark backgrounds, whereas bright spots correspond to atomic columns [2]. High‐angle ADF (HAADF) imaging further enables *Z*‐contrast imaging, allowing for the visualization of lattice structures and chemical compositions at the atomic scale [3]. However, at lower scattering angles (below 60 mrad at 120 kV), the ADF image intensity is no longer dominated only by Z‐contrast. Instead, it becomes highly sensitive to strain‐ and defect‐induced de‐channeling effects of electron beams, which can arise from structural defects (e.g., dislocations and grain boundaries) or point defects (e.g., dopants and vacancies) [4, 5, 6, 7].

High contrast in ADF images is particularly significant in strongly correlated oxides, where strain and defects that induce de‐channeling can substantially affect physical properties [8, 9, 10, 11]. In particular, oxygen vacancies—key ionic defects in oxides—affect local structural distortion and charge doping, significantly altering the physical properties of oxide materials [11, 12, 47]. Although oxygen ions are not directly visualized in ADF images due to their low atomic number, the presence of oxygen vacancies manifests as bright contrast regions, as they enhance the de‐channeling of the electron beam [4, 13, 14]. Therefore, understanding the origins of exceptionally high contrast in ADF imaging beyond conventional *Z*‐contrast is essential for elucidating the fundamental properties of correlated oxides. Recent studies have reported interlayer high contrast in ADF images of TiO_2_/VO_2_ heterostructure systems [15, 16, 17, 18]. These heterostructures serve as a versatile platform for tuning metal–insulator transition (MIT) properties through strain engineering, interfacial effects, and precise control of layer thickness [16, 19, 20, 44, 45]. Specifically, spinodal decomposition—a process in which spontaneous atomic diffusion leads to phase separation from a uniform solution—has been leveraged to fabricate high‐quality heterostructure [15, 17, 18, 21, 22]. In the self‐assembled (Ti,V)O_2_ heterostructure, V‐rich layers exhibit distinctively bright contrast in ADF images [15, 17, 18]. Given the nearly identical atomic numbers of Ti (*Z* = 22) and V (*Z* = 23), this enhanced contrast suggests the presence of additional scattering mechanisms beyond conventional *Z*‐contrast, possibly linked to electron scattering effects associated with atomic disorder.

This study aims to investigate the accumulation of oxygen vacancies in V‐rich layers within self‐assembled (Ti,V)O_2_ heterostructure by considering two key factors: (1) prior research has shown that the chemical potential difference across the TiO_2_/VO_2_ heterointerface stabilizes oxygen vacancy migration from TiO_2_ into VO_2_ layers [14, 23, 24], and (2) the TiO_2_/VO_2_ interface is oriented along the [001]_R_ direction to minimize strain energy from lattice mismatch [18, 21, 22], and thereby aligning the channel pathways along the [001]_R_ known to serve as oxygen ion pathways [23, 25, 26], across the interfaces in the heterostructure. Please note that all orientations not explicitly denoted with subscripts are based on the rutile structure. These structural conditions provide optimal pathways for oxygen vacancies to migrate toward energetically favorable sites during spinodal decomposition. However, detailed analyses of oxygen defects in TiO_2_/VO_2_ heterostructures remain limited, particularly given that the lamellae within these heterostructures span only a few nanometers, necessitating highly localized characterization. Using STEM and electron energy loss spectroscopy (EELS), this study reveals that oxygen vacancies predominantly accumulate within the VO_2_ lamellae of the heterostructure.

## 2 Results and Discussion

**FIGURE 1:**
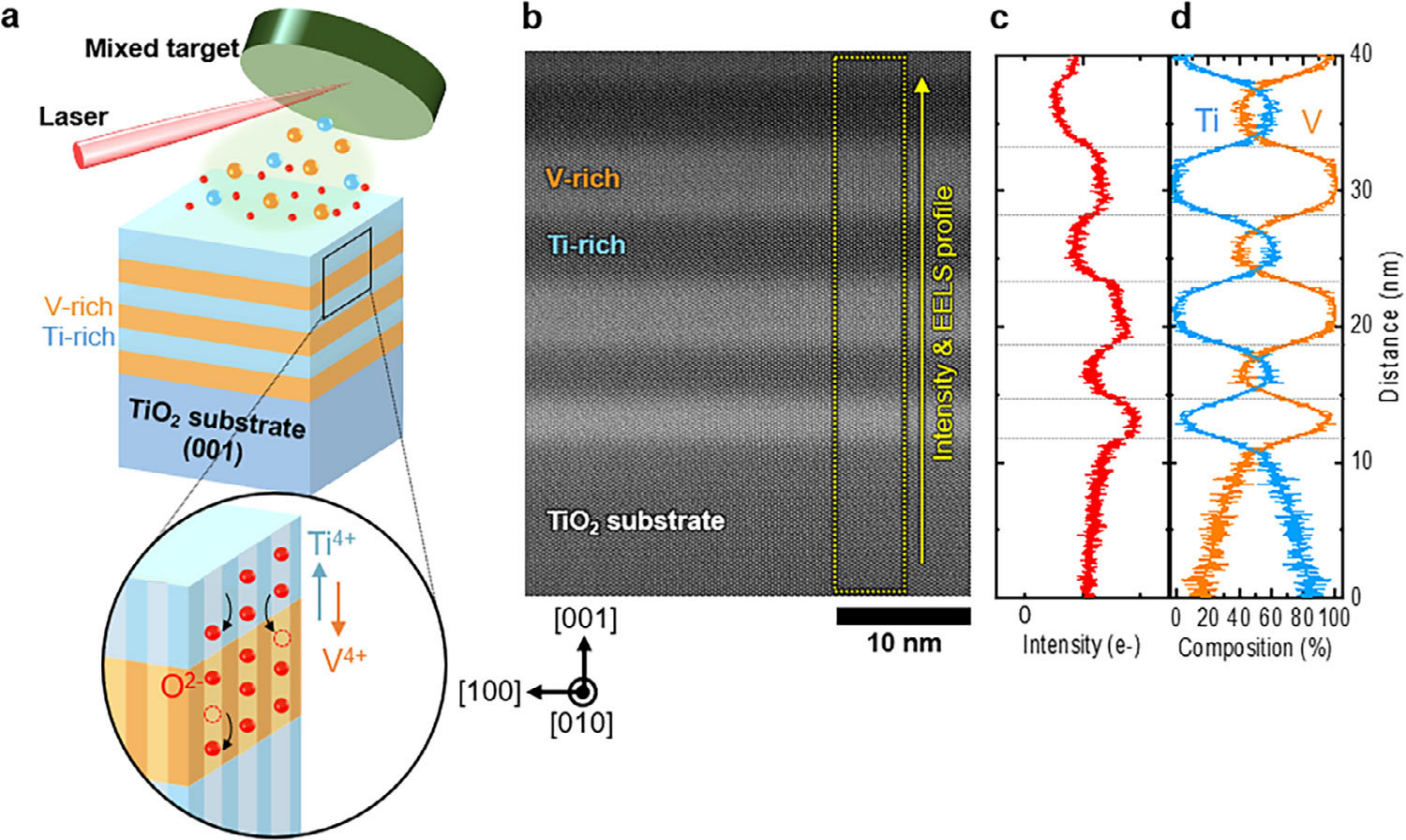
High contrast in ADF‐STEM image of self‐assembled (Ti,V)O_2_ heterostructure on TiO_2_ (001) substrate. (a) Schematic of self‐assembled of (Ti,V)O_2_ heterostructure through spontaneous ionic diffusion during pulsed laser deposition growth. Phase separation results in interface formation on the (001) plane to minimize the strain energy, leading to the generation of a [001]_R_ channel structure across the interface, which facilitates the migration of oxygen ions. (b) Low‐magnification ADF‐STEM image of heterostructure with [010] zone‐axis. Note that the crystallographic indices are expressed with respect to the rutile structure. (c) Intensity profile (red lines) and (d) EELS composition profile (blue and orange lines), showing bright and dark contrast lamellae corresponding to V‐rich and Ti‐rich layers, respectively.

Epitaxial (Ti,V)O_2_ heterostructures are grown on (001) TiO_2_ substrates using pulsed laser deposition (PLD) with a Ti_0.4_V_0.6_O_2_ mixed target. During growth at 420°C, spontaneous phase separation occurs owing to thermally activated atomic diffusion [15, 18, 21], leading to sharp interfaces between the Ti‐rich and V‐rich layers oriented along the [001] direction (Figure 1a). ADF‐STEM imaging of the heterostructure acquired at 120 kV with an ADF detector collection semi‐angle range of 50–160 mrad (Figure S1), reveals high contrast between these layers (Figure 1b). The line profile of ADF intensity (Figure 1c) and EELS composition analysis (Figure 1d) confirm that the dark‐ and bright‐contrast layers correspond to Ti‐rich and V‐rich phases, respectively. EELS spectra indicate that the approximate compositions of the Ti‐rich and V‐rich layers are Ti_0.6_V_0.4_O_2_ and Ti_0.05_V_0.95_O_2_, respectively. Despite the similar atomic numbers of Ti and V, V‐rich layers exhibit a brighter contrast compared with the Ti‐rich layers and TiO_2_ substrate. This observation suggests that, under the present ADF collection semi‐angle range (50–160 mrad), the simultaneous detection of low‐angle scattering signals allows strain or defect related effects to induce electron beam de‐channeling, thereby enhancing electron scattering and contributing to an increased ADF intensity. In contrast, under HAADF conditions, where predominantly high‐angle scattering is collected the contrast largely disappears (Figure S2), indicating that additional contributions beyond conventional Z‐contrast dominate the ADF intensity.

**FIGURE 2:**
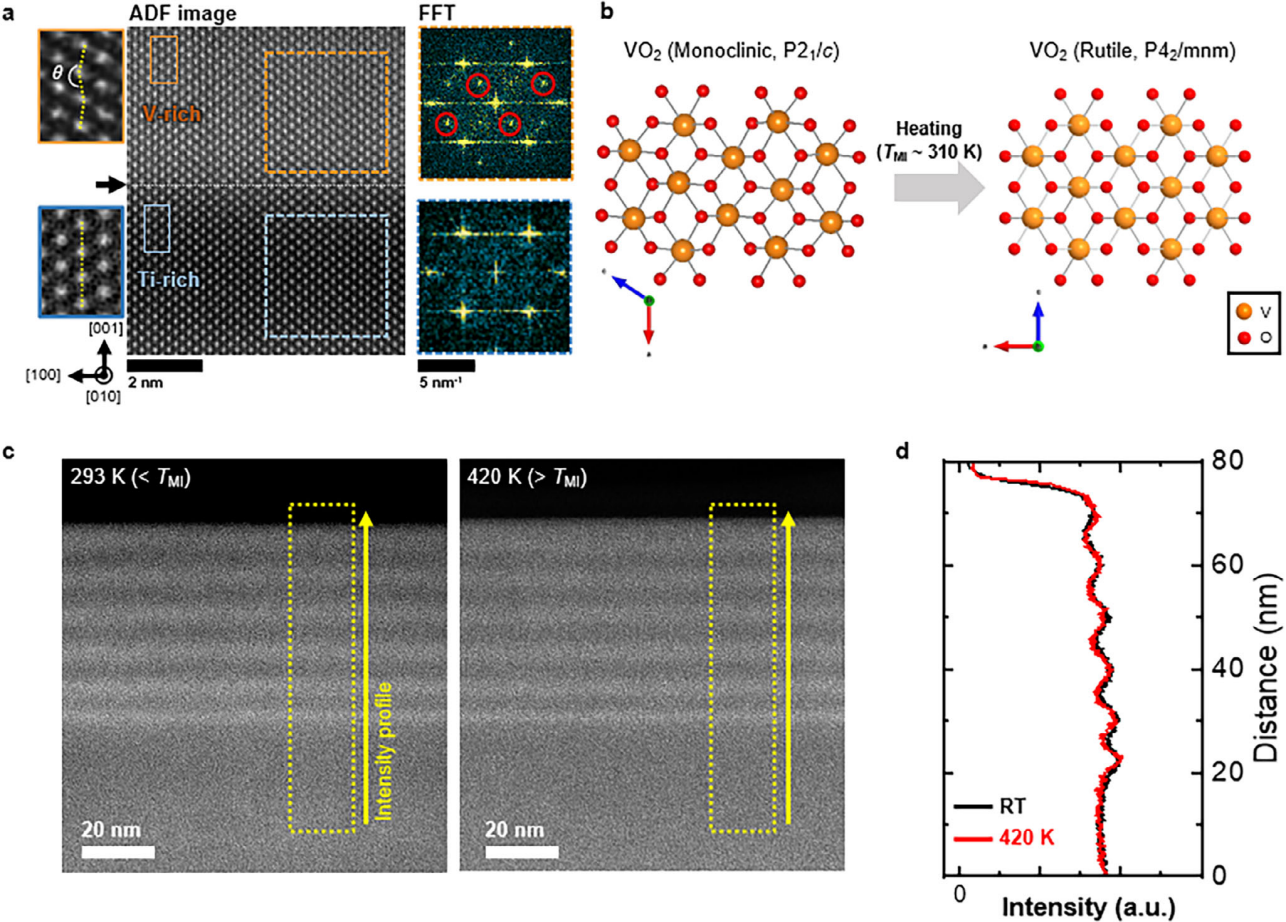
Low‐symmetry in V‐rich layers of (Ti,V)O_2_ heterostructure. (a) ADF images of the V‐rich and Ti‐rich layers, with atomic arrangements corresponding to the orange and blue square regions shown in the left insets. In the monoclinic V‐rich layer, the V─V atomic chains exhibit a zigzag configuration, whereas in the rutile Ti‐rich layer, the Ti─Ti atomic chains are arranged linearly. The FFT patterns obtained from the orange and blue dashed square regions are shown in the dashed boxes on the right. Superlattice peaks (marked by red circles) are observed in the monoclinic V‐rich layer, while the Ti‐rich layer exhibits a high‐symmetry rutile structure. Note that the crystallographic indices are expressed with respect to the rutile structure. (b) Schematic of the thermally driven structural phase transition of VO_2_ from a low‐symmetry monoclinic to a high‐symmetry rutile phase. (c) ADF‐STEM images of heterostructure acquired at 293 K (< *T*_MI_) and 420 K (> *T*_MI_) during in situ heating experiment. (d) Intensity profiles extracted from the ADF images in (c). The bright contrast of the V‐rich layers persists above the *T*_MI_ despite the structural phase transition.

Differences in crystallographic symmetry between the Ti‐rich and V‐rich layers can influence electron scattering and reduce the channeling effect. Figure 2a presents high‐resolution atomic‐scale ADF images of each layer observed along the [010] zone axis with respect to the rutile structure, together with the corresponding fast Fourier transform (FFT) patterns. The FFT pattern obtained from the Ti‐rich layer, indicated by the blue dashed square on the right, exhibits a high‐symmetry rutile structure. In contrast, the FFT pattern from the V‐rich layer, marked by the orange dashed square, shows additional superlattice peaks (highlighted by red circles). The emergence of these superlattice peaks signifies the formation of extended periodicity beyond the fundamental lattice reflecting a lower‐symmetry structure namely the monoclinic phase (Figure S3). The left inset images further show the atomic arrangements of the V‐rich and Ti‐rich regions at higher magnification. In the monoclinic phase, the V─V atomic chains exhibit a zigzag configuration, as indicated by the orange box, whereas in the rutile phase, the Ti─Ti atomic chains remain linearly aligned, as shown by the blue box. Accordingly, quantitative analysis of the tilt angles of the V─V or Ti─Ti atomic chains enables clear phase identification. In the rutile structure, the tilt angle remains constant at 180°, whereas in the monoclinic structure, the tilt angles alternate between positive (θ − 180° > 0) and negative (θ − 180° < 0) values in a regular manner. The experimentally obtained tilt‐angle mapping results for the Ti‐rich and V‐rich layers are consistent with the simulation results, and the detailed data are provided in Figure S4.

When viewed along the electron‐beam direction parallel to the (001) heterostructure interface, the rutile TiO_2_ layer exhibits well‐aligned Ti and O atomic columns, indicating a high degree of crystallographic symmetry. In contrast, the monoclinic VO_2_ layer displays a zigzag arrangement of V─V atomic chains, reflecting a relatively lower symmetry (Figure 2b, left). Such low crystallographic symmetry can disrupt electron trajectories, thereby inducing a de‐channeling effect.

We conducted in situ heating experiments to assess the influence of this low‐symmetry structure on contrast variations in ADF images. The heterostructure undergoes an abrupt transition from an insulation to a metallic state at approximately 310 K [15], during which the V‐rich layers are expected to transition from a monoclinic to a rutile structure (Figure 2b; Figure S5). However, when the heterostructure is heated from 293 to 420 K, well above the transition temperature no significant changes emerge in the bright contrast of the V‐rich layers (Figure 2c) or their intensity profiles (Figure 2d). This result is also reproduced in the simulations (Figure S6), further indicating that the structural phase difference between the Ti‐rich and V‐rich layers is unlikely to be the primary cause of the bright contrast in the ADF images. Instead, other factors, such as strain or ionic defects, are likely responsible for the enhanced contrast in the V‐rich layers.

**FIGURE 3:**
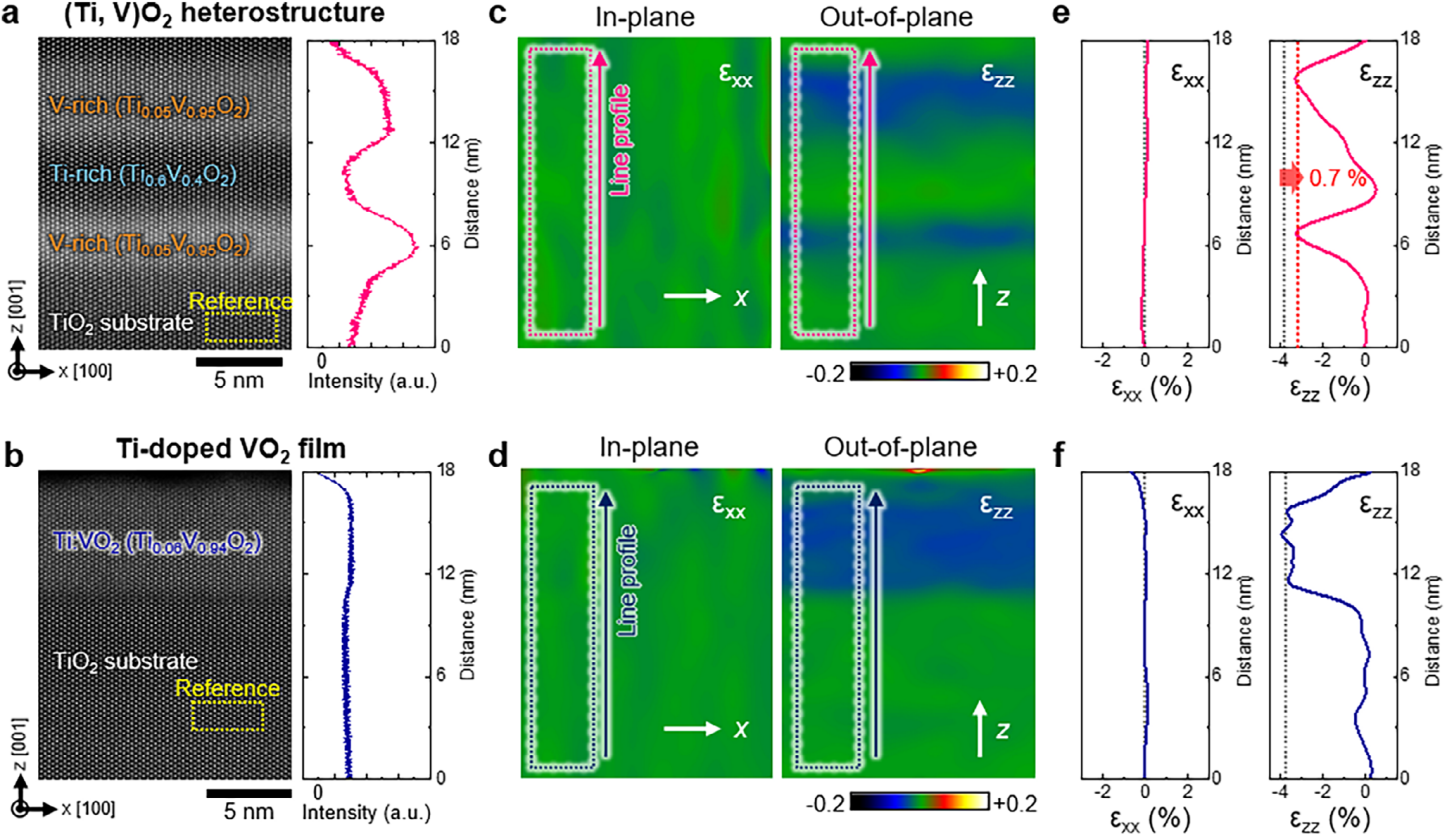
ADF contrast and local strain maps for the (Ti,V)O_2_ heterostructure and Ti‐doped VO_2_ film. ADF image and intensity profiles for (a) V‐rich layers (Ti_0.05_V_0.95_O_2_) in the (Ti,V)O_2_ heterostructure and (b) single‐layer Ti‐doped VO_2_ film (Ti_0.06_V_0.94_O_2_) on TiO_2_ (001) substrates. Relative to the TiO_2_ substrate, the V‐rich layers in the (Ti, V)O_2_ heterostructure exhibit higher intensity, whereas the Ti‐doped VO_2_ film displays nearly uniform intensity despite a similar Ti concentration. Lattice strain mapping of the (c) heterostructure and (d) Ti‐doped VO_2_ film along the in‐plane (*ɛ*_xx_ = (*a* − *a*_substrate_)/*a*_substrate_) and out‐of‐plane (*ɛ_zz_* = (*c* − *c*_substrate_)/*c*_substrate_) directions. The strain values were calculated from the ADF‐STEM images shown in (a) and (b) respectively, using the geometric phase analysis technique. The reference region (yellow dotted box in (a) and (b)) is set on the TiO_2_ substrate. Line profiles of *ɛ*_xx_ and *ɛ*_zz_ extracted from the lattice strain maps of the (e) (Ti,V)O_2_ heterostructure (pink lines) in (c) and (f) Ti:VO_2_ film (navy dashed lines) in (d). The gray dotted lines in the ɛ_zz_ line profiles indicate the reference ɛ_zz_ values calculated from the bulk lattice spacings of VO_2_ and TiO_2_. Note that the crystallographic indices are expressed with respect to the rutile structure.

Another factor potentially contributing to the de‐channeling effect in the V‐rich layers of the heterostructure is the epitaxial strain induced by lattice mismatch with the substrate. VO_2_ has a slightly smaller in‐plane lattice constant (∼0.9%) than that of TiO_2_ along the *a*‐ and *b*‐axes, resulting in tensile strain in the V‐rich layers when grown on a TiO_2_ substrate. To investigate the influence of epitaxial strain on the bright contrast of the V‐rich layers, a single‐layer Ti‐doped VO_2_ (Ti_0.06_V_0.94_O_2_, referred to as Ti:VO_2_) film is deposited on a TiO_2_ (001) substrate for comparison (Figure 3; Figure S7). Ti:VO_2_ adopts the rutile structure with the [010] zone axis (MIT temperature ∼290 K, Figure S5). The Ti:VO_2_ film has a stoichiometry nearly identical to that of the V‐rich layers in the heterostructure. Interestingly, although the V‐rich layer in the heterostructure exhibits significantly higher intensity than that of the TiO_2_ substrate (Figure 3a), the Ti:VO_2_ film shows an intensity nearly identical to that of the TiO_2_ substrate (Figure 3b). To determine whether both films experience identical epitaxial strains from the TiO_2_ substrate, we performed a geometric phase analysis (GPA) to examine the in‐plane (*ε*_xx_) and out‐of‐plane (*ε*_zz_) lattice strains on both films (Figure 3c–f). The GPA strain maps derived from the lattice spacing data extracted from the ADF‐STEM images (Figure 3a,b) represent the relative lattice mismatch with the TiO_2_ substrate—used as a reference (i.e., *ɛ*_xx_ = (*a* − *a*_substrate_)/*a*_substrate_ and *ɛ*_zz_ = (*c* − *c*_substrate_)/*c*_substrate_) [27, 28]. The *ε*_xx_ maps for both the heterostructure and Ti:VO_2_ film appear uniformly green (Figure 3c,d, left), with the *ε*_xx_ line profiles remaining consistently near zero (Figure 3e,f, left). This outcome confirms that both films are fully constrained by the TiO_2_ substrate and experience identical in‐plane strain.

A key difference between the V‐rich layer in the heterostructure and Ti:VO_2_ film emerges in the out‐of‐plane strain (*ε*_zz_) maps. Although both the V‐rich layer and Ti:VO_2_ film appear blue in the *ε*_zz_ color maps owing to their smaller lattice spacing relative to TiO_2_ (Figure 3c,d, right), the *ε*_zz_ line profiles reveal a clear disparity (Figure 3e,f, right).

The expected reference *ε*_zz_ values, calculated using bulk lattice spacing based on Vegard's law [29] and Poisson's ratio [10, 30], are −3.8% for the heterostructure V‐rich layer and −3.7% for the Ti:VO_2_ film (detailed in Supplementary Text S1). Although the measured *ε*_zz_ of the Ti:VO_2_ film closely matches its reference value (Figure 3f, right), the measured *ε*_zz_ of the V‐rich layer in the heterostructure is −3.1%, which is ∼0.7% larger than expected (Figure 3e, right). This finding suggests that, compared with the Ti:VO_2_ film, which shares the same stoichiometry and substrate, the bright contrast in the V‐rich layers of the heterostructure is strongly correlated with out‐of‐plane lattice expansion. This lattice expansion is a well‐established signature of oxygen vacancy formation in VO_2_ [14, 25, 31]. Notably, the 0.7% expansion observed in the heterostructure is consistent with previous reports of oxygen vacancies in VO_2−δ_ films, which resulted in a ∼0.8% expansion [14, 23]. Thus, the GPA strain evaluation strongly supports the presence of oxygen vacancies in the V‐rich layers of the heterostructure, which likely contribute to the observed bright contrast in ADF‐STEM images.

**FIGURE 4:**
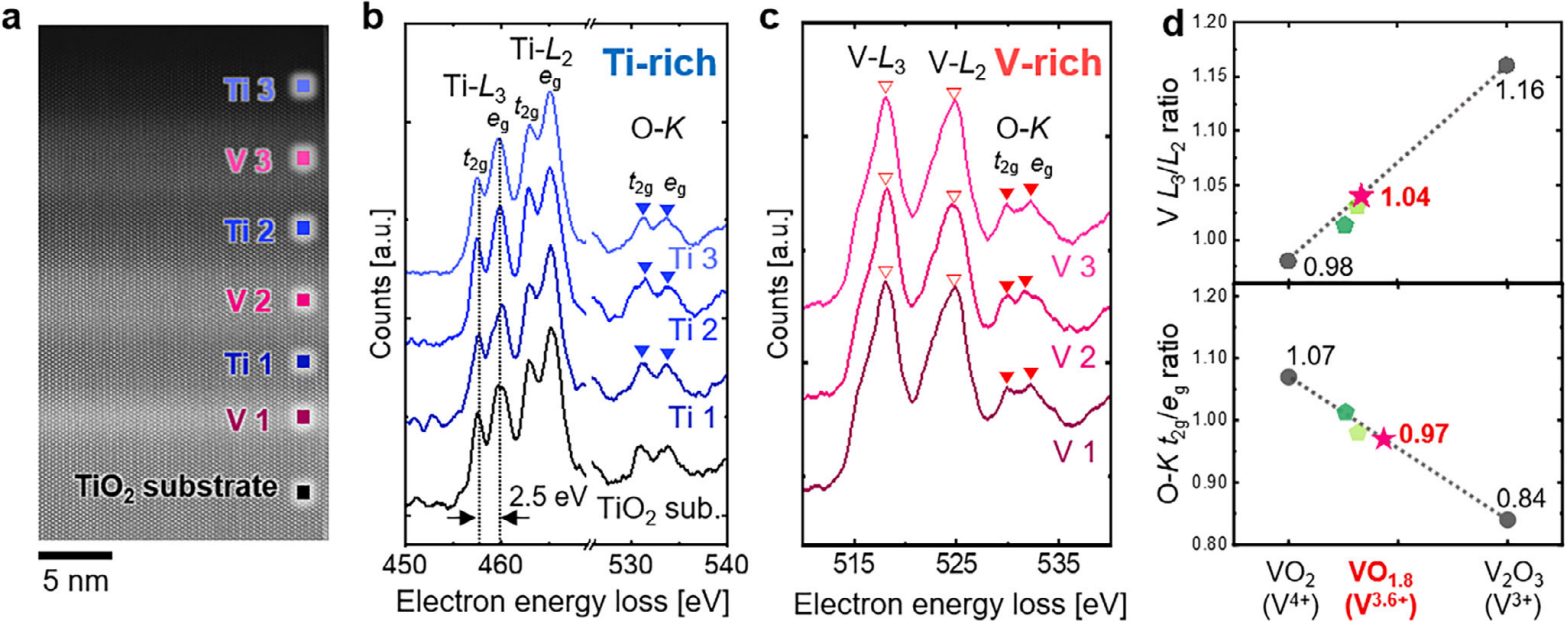
Analysis of local valence state in the (Ti,V)O_2_ heterostructure using EELS. (a) ADF‐STEM image of the (Ti,V)O_2_ heterostructure. EELS spectra are obtained across the heterostructure along the out‐of‐plane direction. (b) Ti *L*_2,3_‐edge and O *K*‐edge spectra obtained from the Ti‐rich layers (Ti 1, Ti 2, Ti 3, marked by blue dots) and TiO_2_ substrate. All spectra from Ti‐rich layers show clear *t*_2g_–*e*_g_ peak splits in Ti *L*_2,3_‐edges and O *K*‐edges, identical to those of the TiO_2_ substrate, indicating the Ti^4+^ valence state with negligible oxygen vacancies. (c) Individual V *L*_2,3_‐edge and O *K*‐edge spectra from the three V‐rich layers (V 1, V 2, and V 3, marked by pink dots). (d) Comparison of the measured values in the V‐rich regions (averaged intensity ratios from the EELS spectra of the V 1, V 2, and V 3 regions, marked by a red star) with the reference values (VO_2_ and V_2_O_3_, marked by gray circles) for the V *L*_3_ */L*_2_‐edge and O *K*‐edge *t*_2g_/*e*_g_ intensity ratios [23, 36]. The light green and dark green pentagons represent the V *L*_3_/*L*_2_‐edge and O *K*‐edge *t*_2g_/*e*_g_ intensity ratios of VO_2_ with 8% and 6.5% oxygen vacancies, respectively as calculated in our previous study [23].

To investigate the distribution of oxygen vacancies within the heterostructure, we performed EELS line scans across multiple Ti‐rich and V‐rich layers. Specifically, Ti *L*_3,2_‐ and O *K*‐edges are measured in three Ti‐rich layers (labeled Ti 1, Ti 2, and Ti 3 in Figure 4a), and V *L*_3,2_‐ and O *K*‐edges are analyzed in three V‐rich layers (labeled V 1, V 2, and V 3 in Figure 4a). These EELS edges are highly sensitive to changes in the valence states of Ti and V, which transition from 4^+^ to 3^+^ in the presence of oxygen vacancies [32, 33, 34, 35, 36, 37, 38]. By comparing these spectral features, the localization of oxygen vacancies within the heterostructure can be determined.

In the Ti‐rich layers, the Ti *L*_3,2_‐ and O *K*‐edges exhibit distinct peak splitting into *t*_2g_ and *e*_g_ components owing to crystal field splitting (Figure 4b), which occurs when Ti ions are surrounded by six oxygen ions [39, 40]; this *t*_2g_–*e*_g_ splitting weakens in the presence of oxygen vacancies owing to altered bonding configurations [32, 33, 34]. However, in all Ti‐rich layers, the splitting energy remains at ∼2.5 eV, identical to that of the stoichiometric TiO_2_ substrate [39]. This indicates that the Ti‐rich layers maintain a stable Ti^4+^ valence state, confirming the absence of significant oxygen vacancies. In contrast, the V‐rich layers exhibit spectral features indicative of mixed valence states. To determine the valence states of V in V‐rich layers, we compare their V *L*_3,2_‐ and O *K*‐edges with reported values for VO_2_ (V^4+^) and V_2_O_3_ (V^3+^). Since the V‐rich region consists of three distinct layers (V 1, V 2, and V 3) the average intensities of these layers were used as the spectral data (details are provided in Table S2) and are denoted by red stars in Figure 4d. Two key spectral ratios are assessed: the V *L*_3_/*L*_2_‐edge intensity ratio (red open arrows in Figure 4c) and O *K*‐edge *t*_2g_/*e*_g_ intensity ratio (red closed arrows in Figure 4c), both of which systematically change as the valence state of V shifts from V^4+^ to V^3+^ [23, 36] (Figure 4d). The V *L*_3_/*L*_2_‐edge intensity ratio in the V‐rich layers is ∼1.04, higher than that 0.98 for VO_2_ but lower than that 1.16 for V_2_O_3_ (Figure 4d, top). Similarly, the O *K*‐edge *t*_2g_/*e*_g_ intensity ratio is ∼0.97 falling between the reference values of 1.07 for VO_2_ and 0.84 for V_2_O_3_ (Figure 4d, bottom). These spectral trends, i.e., an increased V *L*_3_/*L*_2_‐edge ratio and a decreased O *K*‐edge *t*_2g_/*e*_g_ ratio, are well‐known signatures of oxygen vacancies in VO_2−δ_, indicating the presence of a mixed valence state of V^4+^ and V^3+^ [35, 36, 41]. Notably, calculations based on linear interpolation between the reference spectra of VO_2_ and V_2_O_3_ reveal that the V‐rich layers correspond to a VO_1.8_ composition indicating the presence of approximately 10% oxygen vacancies (detailed in Supplementary Text S2). These observations confirm that oxygen vacancies accumulate in the V‐rich layers of the heterostructure.

Combining the lattice expansion observed in the GPA strain evaluation with the valence state shifts identified in EELS, we conclude that oxygen vacancies are accumulated preferentially at the V‐rich layers. These vacancies introduce localized strain fields that distort the atomic arrangement, leading to a de‐channeling effect on the electron beam, which contributes to the observed bright contrast in ADF images. The preferential accumulation of oxygen vacancies in the VO_2_ layers can be explained based on energetic considerations. Owing to the significantly lower formation energy of oxygen vacancies in VO_2_ compared with that in TiO_2_, oxygen vacancy migration from TiO_2_ to VO_2_ is thermodynamically favorable [14, 23, 24]. Additionally, the heterostructure interface is aligned along the (001) plane [18, 21, 22], intersecting the [001]_R_ channels, which serve as oxygen vacancy migration pathways [23, 25, 26]. This structural configuration likely facilitates the selective accumulation of oxygen vacancies in the VO_2_ layers, reinforcing their role in the observed contrast variations in ADF‐STEM images.

## 3 Conclusion

We investigated the origin of the high‐intensity contrast observed in ADF‐STEM images of (Ti,V)O_2_ heterostructure, to unveil the unexpected high contrast in the V‐rich layers. As the ADF image intensity is influenced not only by Z‐contrast but also by elastic scattering effects induced by strain or defects, which can be captured through the adjustment of the collection angle to include low scattering signals. Since the atomic numbers of Ti (Z = 22) and V (Z = 23) are nearly identical, it was expected that the Ti‐rich and V‐rich layers would exhibit similar contrast. However, the high contrast observed in the V‐rich layers is likely due to factors beyond Z‐contrast, such as lower crystallographic symmetry, epitaxial strain, or ionic defects, which may induce electron beam de‐channeling. We found that neither the low symmetry of the monoclinic phase nor the epitaxial tensile strain from the TiO_2_ substrate significantly contributed to the observed contrast enhancement; instead, the abnormal contrast originates from the oxygen vacancies accumulated preferentially at the V‐rich layers. This accumulation of oxygen vacancies is due to oxygen transport along the crystallographic [001]_R_ channels during spinodal decomposition, where spontaneous ionic diffusion drives phase separation. Our study highlights the critical role of these channels in oxygen vacancy migration within rutile‐based oxides, particularly in (Ti,V)O_2_ heterostructure. Given that oxygen vacancies strongly influence the MIT properties of VO_2_, their formation and distribution must be carefully managed in heterostructure systems. Our findings provide valuable insights into the defect engineering of correlated oxides, emphasizing the importance of oxygen vacancy control in optimizing the electronic and structural properties of VO_2_‐based heterostructures for future applications.

## 4 Experimental Methods

### 4.1 Growth of Self‐Assembled (Ti,V)O_2_ Heterostructure

Epitaxial Ti‐rich/V‐rich (Ti,V)O_2_ heterostructure with a thickness of 40 nm were grown on (001) TiO_2_ substrates using PLD (Coherent COMPex Pro 102F) to ablate homogeneously mixed targets. The stoichiometric targets for PLD growth were prepared by sintering dry‐milled stoichiometric powders of V_2_O_5_ (99.99%, Sigma–Aldrich) and TiO_2_ (99.95%, Sigma–Aldrich) at 600°C for 6 h. The composition of the target Ti_0.4_V_0.6_O_2_ was analyzed through energy‐dispersive x‐ray spectroscopy using a high‐resolution field‐emission scanning electron microscope (JSM 7401F, JEOL). The (001) TiO_2_ single‐crystal substrates (Shinkosha) were loaded onto the substrate holder in the PLD chamber, which was evacuated to a base pressure of ∼5 × 10^−7^ Torr. Subsequently, the rotating target was ablated using a KrF excimer laser (λ = 248 nm) with a fluence of 1 J/cm^2^ and a pulse repetition rate of 2 Hz. The growth was performed at a fixed oxygen partial pressure of ∼10 mTorr and substrate temperature of 420°C. After growth, the sample was cooled to 20°C at a rate of 20°C/min.

### 4.2 STEM Analysis

For the atomic‐scale STEM and EELS analyses, cross‐sectional TEM lamellar samples of (Ti,V)O_2_ heterostructure on TiO_2_ substrates were prepared by mechanically grinding samples to a thickness of ∼70 µm, followed by dimpling to a thickness of ∼10 µm and ion milling using an Ar+ ion beam. ADF‐STEM analysis was performed using an aberration‐corrected JEM‐2100F (JEOL) microscope (CEOS GmbH) operated at 120 kV with a probe convergence semi‐angle of 15 mrad and a camera length of 10 cm. The collection semi‐angles were set as 50–160 mrad for ADF imaging to ensure that the ADF contrast reflected not only the HAADF but also the low‐angle ADF (LAADF) signals, which are highly sensitive to strain fields induced by point defects [4, 42].

The raw images were subjected to radial difference filtering to remove background noise (Filters Lite, HREM Research Inc.). For the in situ heating experiments, a Gatan 652 double‐tilt heating holder was used. The lattice strain maps were obtained through GPA from high‐resolution HAADF‐STEM images using the GPA plug‐in (HREM Research Inc.) implemented in Digital Micrograph (Gatan Inc.). The *x*‐ and *y*‐axis orientations were defined as 0° and 90° relative to the horizontal in the images. The reference region was defined within the (001) TiO_2_ substrate regions with known lattice parameters. For chemical analysis, an EELS line scan was performed using an EEL spectrometer (GIF Quantum spectrometer, Gatan) with an energy dispersion range of 0.05 eV and dual EELS mode for the Ti *L*‐ and V *L*‐edges.

## Conflicts of Interest

The authors declare no conflict of interest.

## Supporting information




**Supporting File**: smll73218‐sup‐0001‐SuppMat.docx.

## Data Availability

The data that support the findings of this study are available from the corresponding author upon reasonable request.
