# Supplementary material for: Vacancy‐Induced Z‐Contrast Anomaly in Self‐Assembled (Ti,V)O2 Heterostructure
Source: Small. 2026 Mar 30;22(28):e13789. doi: 10.1002/smll.202513789 (PMC13181509; doi:10.1002/smll.202513789)
Supplement: Supplementary file 1 — Supporting File: smll73218‐sup‐0001‐SuppMat.docx. [file SMLL-22-e13789-s001.docx]

Supporting Information

Vacancy-induced Z-Contrast Anomaly in Self-Assembled (Ti,V)O_2_ heterostructure

Hyeji Sim^†^, Seung-Hyun Heo^†^, Gyung-Min Park^†^, Gi-Yeop Kim, Jaeseoung Park, Junwoo Son, and Si-Young Choi^*^


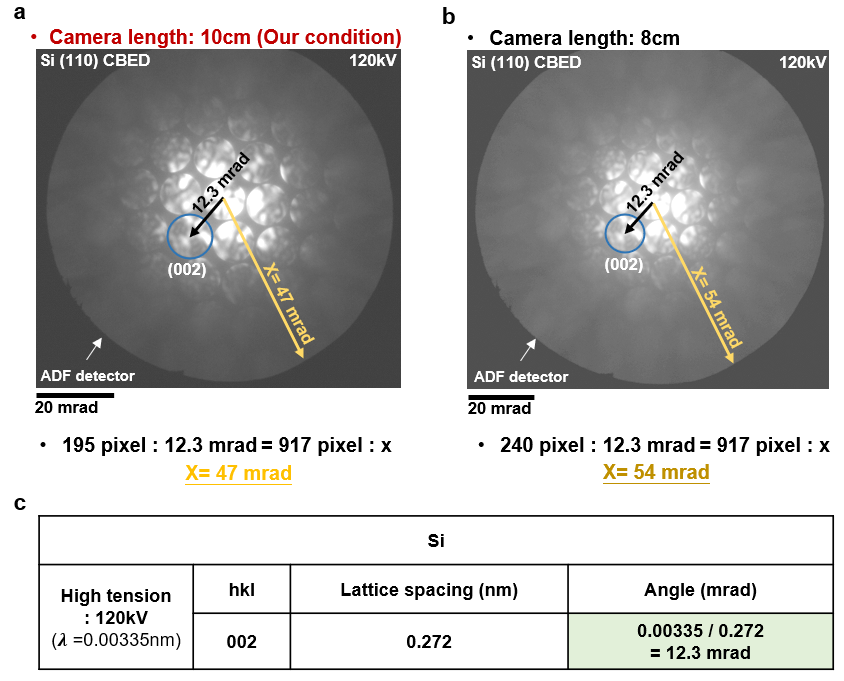


**Figure S1.** ADF collection angle measurement using CBED of a Si (110) single crystal sample (High tension: 120kV) **a)** In the case of camera length 10cm, the boundary indicated by the white arrow represents the ADF detector, and the blue circle corresponds to the diffraction disc of the Si (002) plane. The pixel distance from the transmitted beam to the respective plane is marked with a black arrow. The ADF inner collection angle was measured using the pixel-to-mrad ratio, yielding a value of 47mrad (indicated by the yellow arrow). **b)** In the case of camera length 8cm, the information remains the same as in (a), and the ADF inner collection angle was measured using the pixel-to-mrad ratio, yielding a value of 54mrad (indicated by the yellow arrow). **c)** The angle at which the diffraction disc of the (002) plane scatters from the transmitted beam was theoretically calculated.


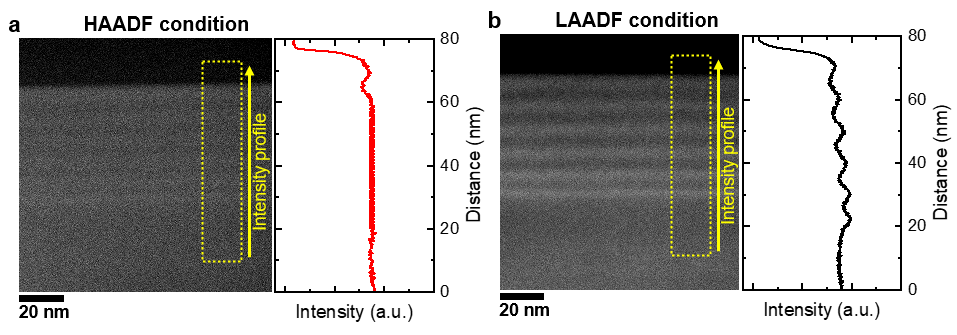


**Figure S2. ADF images of the (Ti,V)O_2_ heterostructure under HAADF and LAADF conditions, respectively. a)** Under HAADF conditions, the intensity profile (indicated by the yellow rectangular box) shows almost no contrast difference. **b)** Under conditions including LAADF signals for the same region, the intensity profile (indicated by the yellow rectangular box) shows a significant contrast difference.


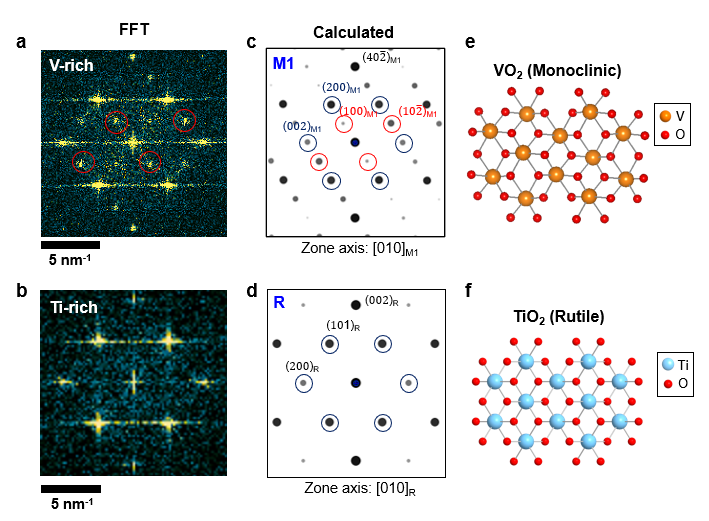


Figure S3. Fast Fourier Transform (FFT) patterns were obtained from the a) V-rich layer and b) Ti-rich layer, which are denoted by orange and blue squares, respectively, in Figure 2a. FFT patterns of the V-rich layer and Ti-rich layer correspond to calculated electron diffraction patterns of c) monoclinic VO_2_ and d) rutile TiO_2_. Atomic structures of e) monoclinic VO_2_ show lower symmetry compared with the f) high-symmetry rutile TiO_2_.

**
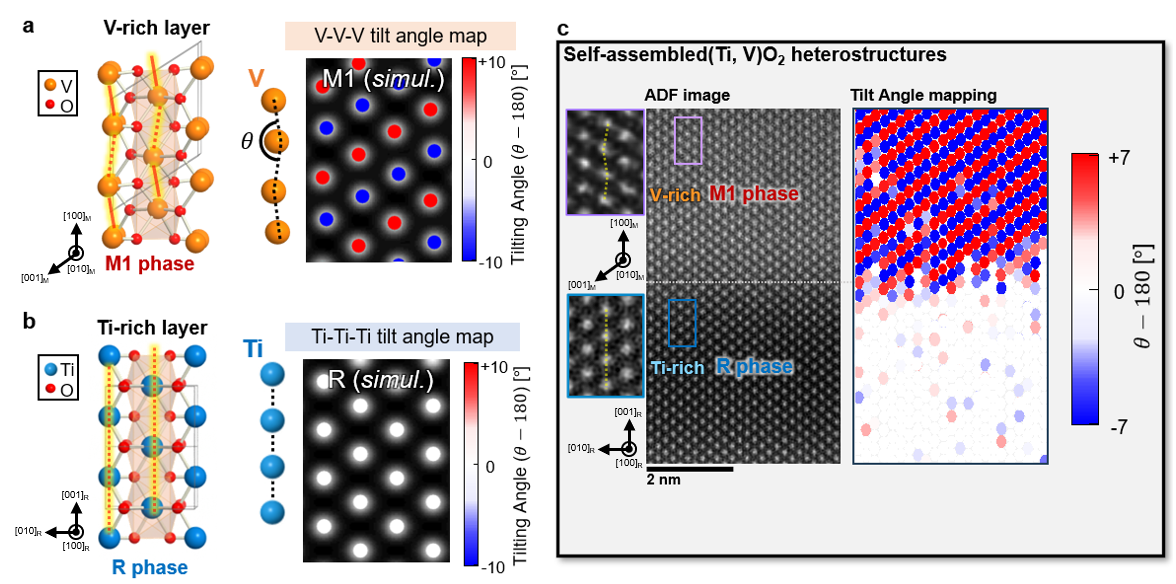
**

**Figure S4.** Atomic-scale structural analysis of the Ti-rich and V-rich layers. **a)** Crystal structure and simulation results of monoclinic VO_2_ along the [010]_M_ zone axis. It is noted that [100]_M_ and [010]_M_ correspond to [001]_R_ and [100]_R_ respectively, in terms of the epitaxial relationship. The V–V atomic chains exhibit a zigzag configuration, and the corresponding tilt-angle mapping shows the tilt angles alternate between positive (θ − 180° > 0) and negative (θ − 180° < 0) values in a regular manner. **b)** Rutile TiO_2_ along the [100]_R_ zone axis. The Ti–Ti atomic chains are arranged linearly, and the buckling-angle mapping shows a nearly uniform angle of approximately 180°. **c)** In the experimental ADF images of the self-assembled (Ti,V)O₂ heterostructure, the V-rich layers exhibiting a monoclinic phase show V–V atomic chains arranged in a zigzag configuration (purple boxes). In contrast, the TiO_2_ layers with a rutile phase (Ti-rich layers) display Ti–Ti atomic chains aligned in a linear manner (blue boxes). This structural distinction is consistently reflected in the tilt-angle mapping results, where the V-rich layers exhibit alternating tilt angles of approximately ±7°, whereas the Ti-rich layers maintain tilt angles close to 0°.


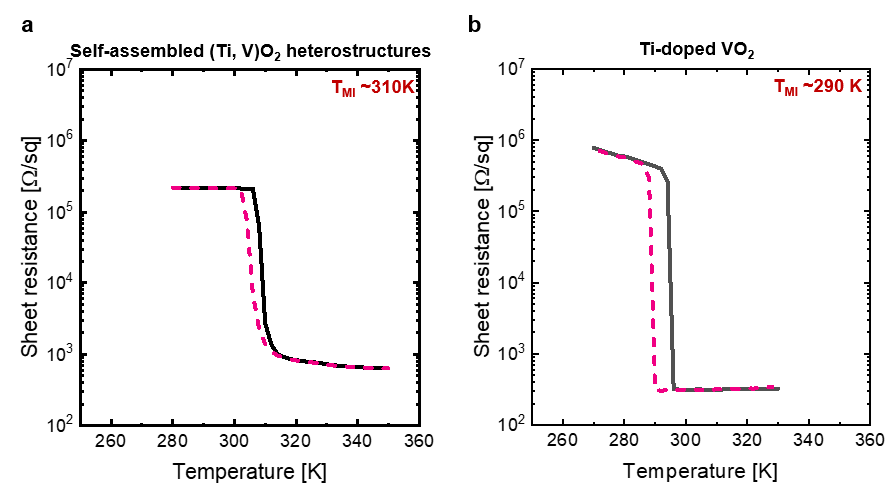


**Figure S5.** **a)** Metal-insulator transition characteristics of the self-assembled (Ti, V)O_2_ heterostructures. The temperature-dependent sheet resistance (R_S_) of the (Ti, V)O_2_ heterostructure, where the black solid line represents the MI characteristics upon heating, and the red dashed line indicates the MI characteristics during cooling. **b)** Metal-insulator transition (MIT) characteristics of the Ti-doped VO_2_ film, showing a metal–insulator transition temperature (T_MI_) of 290 K.


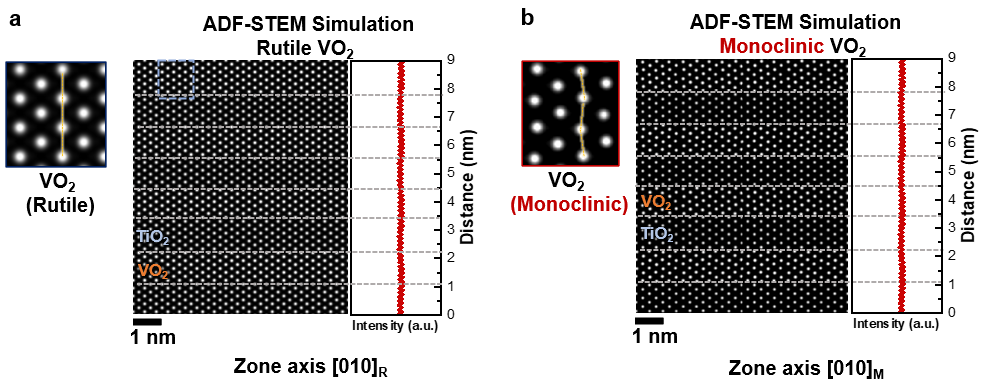


**Figure S6. a)** STEM simulation of the (Ti,V)O_2_ superlattice performed under the rutile VO_2_ where the region highlighted by the blue box corresponds to the rutile VO_2_ layer. In the rutile structure, the V–V atomic arrangement remains nearly linear, with V–V bond angles of approximately 180°. **b)** STEM simulation of the (Ti,V)O_2_ superlattice performed under the monoclinic VO_2_ condition, where the region highlighted by the red box corresponds to the monoclinic VO_2_ layer. In the monoclinic structure, the V–V bond angles deviate from 180°, resulting in a zigzag V–V atomic arrangement.


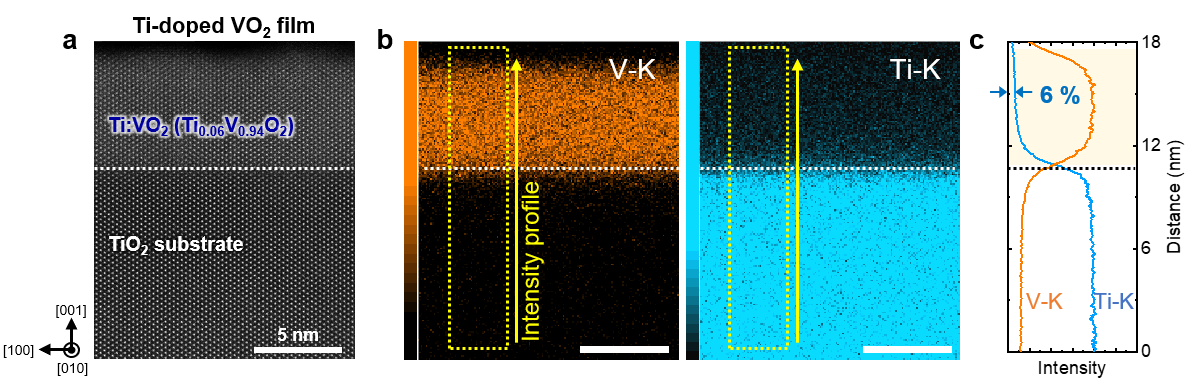


Figure S7. EDS elemental mapping of the Ti:VO_2_ thin film. a) ADF-STEM image and b) EDS color maps of V-*K* edge (orange) and Ti-*K* edge (blue) signals. c) Quantitative analysis of the intensity profile of V-*K* (orange) and Ti-*K* (blue) signals along the yellow arrow in (b). (Note that the crystallographic indices are expressed with respect to the rutile structure)

Supplementary Texts 1

: The calculation of the reference out-of-plane lattice strain (ε_zz_) in GPA of (Ti,V)O_2_ superlattice film and Ti-doped VO_2_ film.

In the lattice strain analysis of (Ti,V)O_2_ superlattice film and Ti-doped VO_2_ film on TiO_2_ substrate using geometric phase analysis (GPA) in Figure 3, a reference lattice strain along the out-of-plane (ε_zz_) of VO_2_ film with respect to the TiO_2_ substrate is defined as following equation:

$$\boldsymbol{\varepsilon}_{\boldsymbol{zz}}=\frac{\boldsymbol{z}_{\boldsymbol{film}}-\boldsymbol{z}_{\boldsymbol{Ti}\boldsymbol{O}_{\mathbf{2}}}}{\boldsymbol{z}_{\boldsymbol{Ti}\boldsymbol{O}_{\mathbf{2}}}} (\mathbf{1})$$

where z_film_ and z_TiO2_ are out-of-plane lattice parameters of (Ti,V)O_2_ superlattice film and TiO_2_ substrate.

The lattice parameters of the V-rich layers in (Ti,V)O_2_ superlattice and Ti-doped VO_2_ film differ from those of bulk VO_2_ due to Ti dopant and epitaxial stress from the TiO_2_ substrate.

First, the changes in lattice parameters of VO_2_ with Ti (Ti_t_V_1-t_O_2_) dopants due to its larger ionic radius can be calculated using Vegard’s law through the following equation^[1]^:

$$\boldsymbol{a}_{\boldsymbol{T}\boldsymbol{i}_{t}\boldsymbol{V}_{\left( \mathbf{1}-\boldsymbol{t} \right)}\boldsymbol{O}_{\mathbf{2}}}=\left( \mathbf{1}-\boldsymbol{t} \right)\boldsymbol{a}_{\boldsymbol{V}\boldsymbol{O}_{\mathbf{2}}}+\boldsymbol{t}\boldsymbol{a}_{\boldsymbol{Ti}\boldsymbol{O}_{\mathbf{2}}} (\mathbf{2})$$

$$\boldsymbol{c}_{\boldsymbol{T}\boldsymbol{i}_{\boldsymbol{t}}\boldsymbol{V}_{\left( \mathbf{1}-\boldsymbol{t} \right)}\boldsymbol{O}_{\mathbf{2}}}=\left( \mathbf{1}-\boldsymbol{t} \right)\boldsymbol{c}_{\boldsymbol{V}\boldsymbol{O}_{\mathbf{2}}}+\boldsymbol{t}\boldsymbol{c}_{\boldsymbol{Ti}\boldsymbol{O}_{\mathbf{2}}} (\mathbf{3})$$

Lattice parameters of bulk TiO_2_, bulk VO_2_ and calculated lattice parameters of each film using Vegard’s law are shown in Table S1 (i)-(iv). Here, as the (001)-VO_2_ film is in an insulating state at room temperature, the lattice parameters of bulk VO_2_ are based on the monoclinic structure in a pseudo-rutile cell.^[2]^


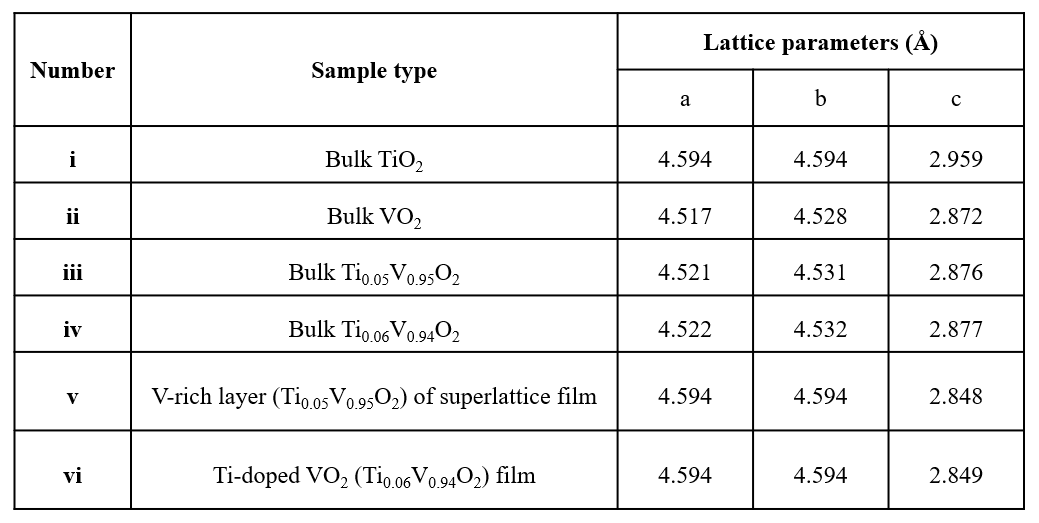


**Table S1.** Lattice parameters of bulk VO_2_ and TiO_2_ and calculated lattice parameters of bulk Ti_t_V_1-t_O_2_ and Ti_t_V_1-t_O_2_ films on TiO_2_ substrate (t = 0.05 in V-rich layers in superlattice film and t = 0.06 in Ti-doped VO_2_ film).

Next, when the epitaxially grown Ti_t_V_1-t_O_2_ films are tightly strained by strained by (001) TiO_2_ substate, the out-of-plane strain due to epitaxial stress from the TiO_2_ substrate can be calculated using Poisson’s ratio of the material through the following equation^[3]^:

$$\boldsymbol{e}_{\boldsymbol{z}}=-\frac{\boldsymbol{\nu}\left( \boldsymbol{e}_{\boldsymbol{x}}+\boldsymbol{e}_{\boldsymbol{y}} \right)}{\mathbf{1}-\boldsymbol{\nu}} (\mathbf{4})$$

The in-plane strains (e_x_ and e_y_) of a film can be defined as the following equations:

$$\boldsymbol{e}_{\boldsymbol{x}}=\frac{\boldsymbol{x}_{\boldsymbol{film}}-\boldsymbol{x}_{\boldsymbol{bulk}}}{\boldsymbol{x}_{\boldsymbol{bulk}}} (\mathbf{5})$$

$$\boldsymbol{e}_{\boldsymbol{y}}=\frac{\boldsymbol{y}_{\boldsymbol{film}}-\boldsymbol{y}_{\boldsymbol{bulk}}}{\boldsymbol{y}_{\boldsymbol{bulk}}} (\mathbf{6})$$

Both (Ti, V)O_2_ superlattice film and Ti-doped VO_2_ film are fully strained both along the in-plane x- and y-direction (Figure 3c, 3d), so a_film_ = a_TiO2_ = 4.594 Å, b_film_ = b_TiO2_ = 4.594 Å^[4]^. With the value ν = 0.249 for VO_2_, the calculated lattice parameters of the (Ti,V)O_2_ superlattice film and Ti-doped VO_2_ film using Poisson’s ratio of the material are shown in Table S1 (v)-(vi).

For the V-rich layer in (Ti,V)O_2_ superlattice film, the calculated in-plane strains using equations (5) and (6) can be obtained as e_x_ = 0.0162, e_y_ = 0.0138. With the values e_x_, e_y_, and ν = 0.249 for VO_2_^[3]^, equation (4) gives the value -0.0100 for out-of-plane strain e_z_, and thus the out-of-plane lattice parameter z_film_ = 2.848 Å. Putting the z_film_ value into the equation (1) with the value z_TiO2_ = c_TiO2_ = 2.959 Å, the reference ε_zz_ of (Ti,V)O_2_ superlattice film in GPA is -0.038 (denoted as gray dashed line in Figure 3e).

For the Ti-doped VO_2_ film, the calculated in-plane strains using equations (5) and (6) can be obtained as e_x_ = 0.0160, e_y_ = 0.0137. With the values e_x_, e_y_, and ν = 0.249 for VO_2_, equation (4) gives the value -0.0101 for out-of-plane strain e_z_, and thus the out-of-plane lattice parameter z_film_ = 2.849 Å. Putting the z_film_ value into equation (1) with the value z_TiO2_ = c_TiO2_ = 2.959 Å, the reference ε_zz_ of Ti-doped VO_2_ film in GPA is -0.037 (denoted as gray dashed line in Figure 3f).

Supplementary Texts 2

: The calculation of oxygen vacancy concentration in the V-rich layers of (Ti,V)O_2_ superlattice film

To quantitatively determine the oxygen vacancy concentration in the V-rich layers of (Ti,V)O_2_ superlattice films we employed spectral indicators obtained from electron energy-loss spectroscopy (EELS) namely the V L-edge and O K-edge. These indicators are highly sensitive to variations in the vanadium oxidation state and therefore exhibit distinct changes depending on the oxygen vacancy concentration.

The V L-edge corresponds to electronic transitions from the V 2p core level to the unoccupied V 3d states. Spin–orbital coupling splits the V 2p level into L_3_ (j = 3/2, fourfold degeneracy) and L_2_ (j = 1/2, twofold degeneracy). Because the L_3_ edge provides a greater number of transition channels its relative intensity becomes more pronounced as the occupancy of the 3d orbitals increases with the formation of oxygen vacancies. Consequently the V L_3_/L_2_–edge intensity ratio exhibits a proportional increase with increasing oxygen vacancy concentration.

The O K-edge reflects transitions from the O 1s core level to unoccupied O 2p states, which are strongly hybridized with V 3d orbitals. In the O K-edge spectrum the t_2g_ and e_g_ peaks correspond to the lower-energy t_2g_ peak (π bonding) and the higher-energy e_g_ peak (σ bonding). While the e_g_ orbitals are located above the band gap and remain unoccupied the t_2g_ states lie near the fermi level and are progressively populated with electrons as the vanadium oxidation state decreases. Consequently the spectral weight of the unoccupied t_2g_ states decreases with increasing oxygen vacancies (corresponding to a reduction in the vanadium oxidation state) and therefore the O K-edge t_2g_/e_g_ intensity ratio decreases inversely with the oxygen vacancy concentration.

Therefore the V L_3_/L_2_-edge ratio and the O K-edge t_2g_/e_g_ ratio can serve as direct indicators of oxygen vacancies. Since the measured values in the V-rich layers lie between the reference standards of VO_2_ (V^4+^) and V_2_O_3_ (V^3+^) their relative positions can be tracked to quantitatively evaluate the oxygen vacancy concentration within the V-rich layers of the (Ti,V)O_2_ superlattice films.

Oxygen vacancy quantification method and calculation

The oxygen vacancies in the V-rich layers were quantified by applying linear interpolation between the reference values of VO_2_ (V^4+^) and V_2_O_3_ (V^3+^).^[5]^ This approach assumes a linear correlation of the V L_3_/L_2_-edge and O K-edge t_2g_/e_g_ ratios with the vanadium oxidation state and estimates the oxygen vacancy concentration using the slope derived from VO_2_ (V^4+^) and V_2_O_3_ (V^3+^). Through this method the average oxidation state of vanadium in the V-rich layers was determined from which the actual VO_x_ composition and the corresponding oxygen vacancy concentration were subsequently obtained.

In the (Ti,V)O_2_ superlattice films, the V L_3_/L_2_-edge intensity ratio and the O K-edge t_2g_/e_g_ ratio in the V-rich layers were measured to be 1.04 and 0.97 respectively. These values were calculated as the averaged intensities (detailed values provided in Table S2) from the V 1, V 2, and V 3 regions shown in Figure 4c and are marked by a red star in Figure 4d.


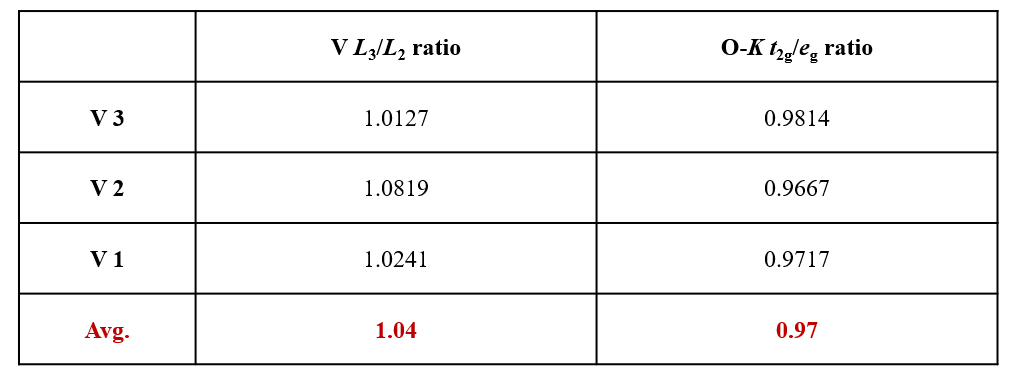


**Table S2.** V L_3_/L_2_-edge and O K-edge t_2g_/e_g_ ratios obtained from three regions (V 1, V 2, and V 3) of the V-rich layer in the (Ti,V)O_2_ superlattice film shown in Figure 4c, together with their averaged values (marked by a bold red).

$$\boldsymbol{VO}_{\boldsymbol{x}}, \boldsymbol{x}= \frac{\boldsymbol{V} \boldsymbol{Oxidation} \boldsymbol{state}}{\mathbf{2}} (\mathbf{1})$$

1. V L_3_/L_2_–edge intensity

$$\boldsymbol{\Delta V} \boldsymbol{oxidation} \boldsymbol{state} =\frac{\boldsymbol{\Delta V} \boldsymbol{L}_{\mathbf{3}}/\boldsymbol{L}_{\mathbf{2}}-\boldsymbol{edge} \boldsymbol{ratio}}{\boldsymbol{V}\boldsymbol{L}_{\mathbf{3}}/\boldsymbol{L}_{\mathbf{2}}-\boldsymbol{edge} \boldsymbol{ratio} \boldsymbol{slope}} (\mathbf{2})$$

The V L_3_/L_2_-edge ratios of VO_2_ (V^4+^) and V_2_O_3_ (V^3+^) were measured to be 0.98 and 1.16 respectively, giving a ΔV_ref_ L_3_/L_2_-edge ratio = 0.18 calculated as (1.16 – 0.98). Since the oxidation state difference between V^4+^ and V^3+^ is one, the V_ref_ L_3_/L_2_-edge ratio slope is 0.18 calculated as (0.18/1). In the V-rich layers of the (Ti,V)O_2_ superlattice the measured V_v-rich_ L_3_/L_2_-edge ratio was 1.04 which is 0.06 (ΔV_v-rich_ L_3_/L_2_-edge ratio) higher than the VO_2_ (V^4+^) reference value (0.98). This difference corresponds to ΔV_v-rich_ oxidation state = 0.33 calculated as (0.06/0.18) as derived from equation (2), resulting is an average valence of V^3.67+^. According to equation (1), this oxidation state translates to a composition of VO_1.83_, indicating the presence of approximately 8.5% oxygen vacancies in the V-rich layers.

2. O K-edge t_2g_/e_g₉_ intensity

$$\boldsymbol{\Delta V} \boldsymbol{oxidation} \boldsymbol{state} =\frac{\boldsymbol{\Delta O} \boldsymbol{K}-\boldsymbol{edge} \boldsymbol{t}_{\mathbf{2}\boldsymbol{g}}/\boldsymbol{e}_{\boldsymbol{g}} \boldsymbol{ratio}}{\boldsymbol{O} \boldsymbol{K}-\boldsymbol{edge} \boldsymbol{t}_{\mathbf{2}\boldsymbol{g}}/\boldsymbol{e}_{\boldsymbol{g}} \boldsymbol{ratio} \boldsymbol{slope}} (\mathbf{3})$$

The O K-edge t_2g_/e_g_ ratios of VO_2_ (V^4+^) and V_2_O_3_ (V^3+^) were measured to be 1.07 and 0.84 respectively, giving a ΔO_ref_ K -edge t_2g_/e_g_ ratio = –0.23 calculated as (0.84 - 1.07). Since the oxidation state difference between V^4+^ and V^3+^ is one, the O_ref_ K -edge t_2g_/e_g_ ratio slope is –0.23 calculated as (-0.23/1). In the V-rich layers of the (Ti,V)O_2_ superlattice the measured O K_v-rich_ -edge t_2g_/e_g_ ratio was 0.97 which is –0.10 (Δ O K_v-rich_ -edge t_2g_/e_g_ ratio) lower than the VO_2_ (V^4+^) reference value (1.07). This difference corresponds to ΔV_v-rich_ oxidation state = 0.434 calculated as (–0.10/–0.23) as derived from equation (3), resulting is an average valence of V^3.56+^. According to equation (1) this value corresponds to a composition of VO_1.78_ indicating the presence of ~11% oxygen vacancies in the V-rich layers.

In our previous study^[6]^, we validated the reliability of this interpolation method by plotting the oxygen vacancy data quantified from EELS spectra. For a (2-2) sample (marked by light green pentagon) with ~8% oxygen vacancies the measured V L₃/L₂-edge and O K-edge t_₂g_/e_g_ ratios were 1.03 and 0.98 respectively. The ΔV oxidation state = 0.32 calculated as (2 × 0.16) as derived from equation (2), resulting is an average valence of V^3.68+^ and the corresponding ΔV L_3_/L_2_-edge ratio was 0.05 calculated as (1.03 – 0.98) compared to the VO_2_ (V^4+^) value (0.98). According to equation (2) this corresponds to a slope of 0.16 calculated as (0.05/0.32) for the V L_3_/L_2_-edge ratio which is within ~10% of the interpolated slope 0.18 (V_ref_ L_3_/L_2_ ratio slope) thereby confirming the reliability of the linear interpolation method. Similarly, the (2-1) sample (marked by dark green pentagon) with ~6.5% oxygen vacancies also followed the expected linear trend.

These results collectively demonstrate that linear interpolation between VO_2_ and V_2_O_3_ reference values provide a reliable framework for estimating oxygen vacancy fractions from EELS spectral indicators. Analysis of the V L_3_/L_2_ edge intensity yields a composition of VO_1.83_ and the O K-edge t_2g_/e_g_ intensity suggests VO_1.78_ in the V-rich layers of the (Ti,V)O_2_ superlattice films. On this basis, we conclude that the V-rich layers in the (Ti,V)O_2_ superlattice films correspond on average to a VO_1.8_ composition, indicating the presence of ~10% oxygen vacancies.


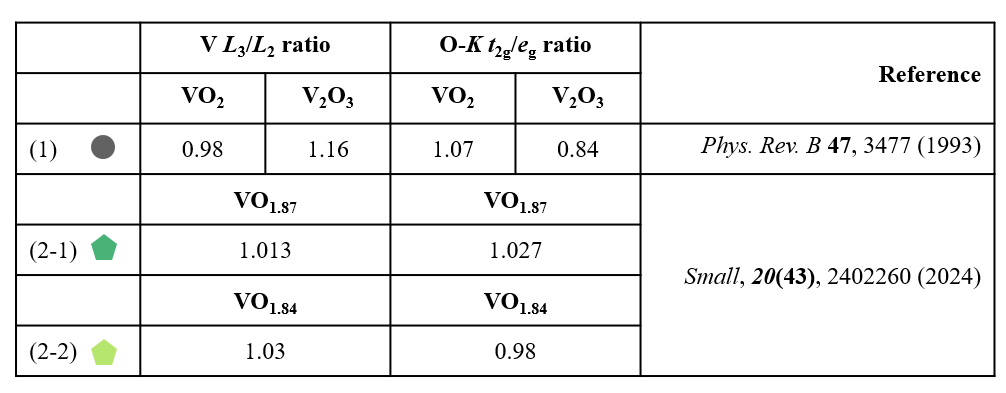


Table S3. Comparison of intensity ratios of V *L*_3_/*L*_2_ -edge and O *K*-edge *t*_2g_/*e*_g_ obtained from VO_2_ and V_2_O_3_ in previous study.^[5]^ The values from each reference are represented by different shapes and colors in the graph of Figure 4d. The reference values of VO_2_ and V_2_O_3_ are marked by gray circles whereas the values of VO_2_ with 6.5% and 8% oxygen vacancies calculated in our previous study^[6]^ are represented by dark green and light green pentagons, respectively.

**References**

[1] L. Vegard, “Die konstitution der mischkristalle und die raumfüllung der atome,” *Zeitschrift für Physik* **1921**, 5, 17.

[2] Z. Hiroi, T. Yoshida, J. Yamaura, Y. Okamoto, “Spinodally decomposed nanostructures in a TiO_2_–VO_2_ crystal.” *APL Materials* **2015**, 3.

[3] N. B. Aetukuri, A. X. Gray, M. Drouard, M. Cossale, L. Gao, A. H. Reid, R. Kukreja, H. Ohldag, C. A. Jenkins, E. Arenholz, “Control of the metal–insulator transition in vanadium dioxide by modifying orbital occupancy,” *Nature Physics* **2013**, 9, 661.

[4] L. Rodríguez, E. Del Corro, M. Conroy, K. Moore, F. Sandiumenge, N. Domingo, J. Santiso, G. Catalan, “Self-pixelation through fracture in VO_2_ thin films,” *ACS Applied Electronic Materials* **2020**, 2, 1433.

[5] X. Lin, Y. Wang, V. Dravid, P. Michalakos, M. Kung, “Valence states and hybridization in vanadium oxide systems investigated by transmission electron-energy-loss spectroscopy,” *Physical Review B* **1993**, 47, 3477.

[6] H. Sim, K. Y. Doh, Y. Park, K. Song, G. Y. Kim, J. Son, D. Lee, S. Y. Choi, “Crystallographic Pathways to Tailoring Metal‐Insulator Transition through Oxygen Transport in VO_2_,” *Small* **2024**, 20, 2402260.
